# Multiple Linear Regression for Reconstruction of Gene Regulatory Networks in Solving Cascade Error Problems

**DOI:** 10.1155/2017/4827171

**Published:** 2017-01-29

**Authors:** Faridah Hani Mohamed Salleh, Suhaila Zainudin, Shereena M. Arif

**Affiliations:** ^1^Department of Software Engineering, College of Computer Science & IT, Universiti Tenaga Nasional, Jalan IKRAM-UNITEN, 43000 Kajang, Malaysia; ^2^Centre of Artificial Intelligence, Faculty of Information Sciences & Technology, Universiti Kebangsaan Malaysia (UKM), 43650 Bangi, Malaysia; ^3^Department of Information Technology, Faculty of Computing and Information Technology in Rabigh, King Abdulaziz University, Rabigh, Saudi Arabia

## Abstract

Gene regulatory network (GRN) reconstruction is the process of identifying regulatory gene interactions from experimental data through computational analysis. One of the main reasons for the reduced performance of previous GRN methods had been inaccurate prediction of cascade motifs. Cascade error is defined as the wrong prediction of cascade motifs, where an indirect interaction is misinterpreted as a direct interaction. Despite the active research on various GRN prediction methods, the discussion on specific methods to solve problems related to cascade errors is still lacking. In fact, the experiments conducted by the past studies were not specifically geared towards proving the ability of GRN prediction methods in avoiding the occurrences of cascade errors. Hence, this research aims to propose Multiple Linear Regression (MLR) to infer GRN from gene expression data and to avoid wrongly inferring of an indirect interaction (A → B → C) as a direct interaction (A → C). Since the number of observations of the real experiment datasets was far less than the number of predictors, some predictors were eliminated by extracting the random subnetworks from global interaction networks via an established extraction method. In addition, the experiment was extended to assess the effectiveness of MLR in dealing with cascade error by using a novel experimental procedure that had been proposed in this work. The experiment revealed that the number of cascade errors had been very minimal. Apart from that, the Belsley collinearity test proved that multicollinearity did affect the datasets used in this experiment greatly. All the tested subnetworks obtained satisfactory results, with AUROC values above 0.5.

## 1. Introduction

The GRN inference-related works have fueled many major breakthroughs in finding drug targets for the treatment of human diseases, including cancer [1–3]. Therefore, being able to predict gene expressions more accurately provides a way to explore how drugs affect a system of genes, as well as for identifying the genes that are interrelated in a process. Besides, rebuilding GRN from gene expression profiles allows the discovery of various functions ranging over diverse domains like molecular biology, biochemistry, bioengineering, and pharmaceutics [2].

One of the main reasons for the reduced performance of previous GRN methods had been inaccurate prediction of cascade motifs. Although there are various gene prediction methods that were developed and presented in various leading journals before, discussion on specific methods of solving problems related to cascade errors is still lacking. The study conducted by [4–11] discussed the issue of cascade errors. However, the experiments conducted were not specifically geared towards proving the ability of GRN prediction methods in avoiding the occurrence of cascade errors. Distinguishing between direct and indirect regulation (cascade errors) is a well-known difficulty in GRN inference but was never quantitatively assessed.

Inferring GRNs remain challenging because of several limitations: (1) the high dimensionality of living cells is where tens of thousands of genes act at different temporal and spatial combinations; (2) one gene or gene product may interact with multiple partners, either directly or indirectly and thus possible relationships are dynamic and nonlinear; (3) current high-throughput technologies generate data that involve a substantial amount of noise  [9, 12]; (4) the sample size is extremely low compared with the number of genes [13, 14] and the presence of hidden nodes [9]. Using the case of a simple cascade *i* → *k* → *j*, when intermediate node *k* is hidden, nodes *i* and *j* become isolated from each other. Then, all indirect paths between them became hidden, hence interrupting the prediction of the whole GRN.

With that, this research aims to propose Multiple Linear Regression (MLR) to infer GRN from gene expression data and to avoid wrongly inferring of an indirect interaction (A → B → C) as a direct interaction (A → C). MLR was selected because MLR takes into account a combination of effects and simultaneous observations. This work is different from other regression analysis-based researches such as [10, 11, 15–18] in a way that it presents novel experimental procedures to assess the effectiveness of GRN inference method in dealing with cascade error. Lastly, this work proposes a novel experimental procedure to assess the effectiveness of MLR in dealing with cascade error. Although MLR achieved an acceptable level of performance when dealing with cascade motifs, two main problems had been detected from our experience in using MLR for GRN inference. The problems are that MLR is unable to process datasets of structure *n* ≤ *p* (*n* = observations and *p* = variables) and does not cater for multicollinearity problem among the predictors.

## 2. Past Researches

Various methods have been applied in GRN construction. We categorize the methods into nine categories. Information-theoretic approach is dominated by methods such as Path Consistency Algorithm based on Conditional Mutual Information [7] and Mutual Information Test based on Dynamic Bayesian Network [19] and Mutual Information [20]. As for filter-based approaches, Unscented Kalman Filter [21] and Fractional Kalman Filter [22] were proposed. Under graph-based category, method such as Random Forests or Extra-Trees [23] was applied. Probability and Statistics category has methods such as Gaussian Graphical Model [24] and Double *t*-test [25]. The emerging algorithms such as Particle Swarm Optimization and Ant Colony Optimization [26] are categorized under nature-inspired category. For the category of correlation and dependence, methods such as Local Expression Pattern [27] and three DC- (Distance Correlation-) based algorithms, CLR-DC, MRNET-DC, and REL-DC [28], were proposed. For machine learning category, Markov Logic network [29] was applied. We purposely categorized the past approaches into a category called hybrid methods. The methods in this category incorporated more than one method such as collaboration of Mutual Information and Regression [30], Ordinary Differential Equation-based Recursive Optimization (RO), and Mutual Information (MI) [12] and Linear Regression combined with Bayesian Model [31].

## 3. Problem Statements

The findings obtained by Salleh et al. [32] pertaining to the topics discussed in this study proved that most of the false positives had been due to cascade errors. Meanwhile, researches conducted by [4, 33] were strongly affected by cascade motifs, where these methods systematically predicted false positive interactions [34]. In addition, studies conducted by [10, 12, 35–37] depicted similar opinion, in which the main source of false positive predictions had been* indirect effects* or* cascade errors*. Apart from the term* cascade error*, other terms, such as* indirect effects*, are also used in the manuscript [10].

Despite the active research on various gene prediction methods, the discussion on specific methods to solve problems related to cascade errors is still lacking. In fact, the experiments conducted by the past studies were not specifically geared towards proving the ability of GRN prediction methods in avoiding the occurrences of cascade errors. Only recently, GNW (GeneNetWeaver), which was developed by [34], has offered tremendous positive impact to the area of systems biology, especially GRN prediction. GNW has been found to provide many features concerning GRN inference performance assessment, including network motifs analysis. However, one problem that hampers the network motifs analysis is that if the GRN inference method was tested by using complex experimental data, the results generated by the GNW would be quite distorted. Thus, the complexity in handling complex data and predicting certain types of genes interactions had motivated the researchers to design, develop, and assess the proposed method towards solving the cascade errors.

## 4. Overview of Data

In this study, real experiment datasets were utilized from M3D [38]. M3D provided manually curated metadata for their chip measurements. The expression data can be obtained from http://m3d.mssm.edu/. The predicted* E. coli* interactions were validated based on gold standard networks of* E. coli* obtained from GNW [34]. There were 4297 genes, with a maximum of 907 chips (observations). Other references that were also used had been obtained from similar datasets, such as those presented by [10, 12, 25].

## 5. GRN Prediction Methods by Using the Regression-Based Technique

In recent years, methods in regression analysis category have received ever increasing attention in the GRN inference research area. The existing research was conducted using the regression models such as Multiple Regression [17], LASSO [15], Ridge Partial Least Squares Regression [16], and ANOVA [10].

Regression analysis is known as a complex math-based method that will take some time to be applied. Nowadays, with many improvements done in certain software, the implementation of regression analysis has been simplified, though not completely. The success of application of regression-based methods on modeling the gene expression and DND microarray data depends on the choice of model and predictors that will be used as the input [15]. Reference [15] proposed a method named GEMULA, which has a four-stage method based on LASSO, used to identify and prioritize the synergistic interaction among predictors. Reference [16] has proposed a new method of identifying genes using Partial Least Squares. The estimation problem has been solved by combining Partial Least Squares Ridge with RFE and error Brier using two-nested CV. Ridge method has been receiving increasing attention from researchers based on its ability to tackle problems related to multicollinearity [39]. One of the main issues that need to be considered in applying the regression analysis is how to make GRN predictions with a limited number of observations. Reference [18] stated that the low number of samples is one of the key issues that need to be addressed. Reference [10] emphasizes the ability of ANOVA to be applied to gene expression data without having to perform nonlinear discretization process. Discretization is the process used to convert a continuous equation into a form that can be used to calculate the numerical solution. Another study is from [17] which aims to improve the accuracy of forecasting large-sized networks. This study uses MLR by applying parallel processing techniques. However, this study was conducted on data already in the ideal state of 1000 × 1000 gene perturbation experiments, which means that the number of observations does not exceed the number of genes. Their algorithm was parallelized to handle large problems in a computationally efficient manner by distributing the overall computational burden among different processors to reduce the total execution time. However, their paper did not explain in detail how the separate predictions were combined to perform the complete prediction for the whole complete set of data at one time. Apart from the study by [10, 11], all of the studies reviewed in this manuscript do not discuss specifics about how to solve the issue of cascade error. The next paragraph specifically explains the researches that cater for cascade motif.

The study from [9] is one of the main researches that serve as benchmarks for the viability of the silencing method in performing GRN prediction to the large network. Reference [9] has proposed several formulas which further highlight the direct relationship between genes versus indirect relationship; hence, prediction of a direct relationship is more easily done without any interference of an indirect relationship. Apart from the effects of indirect relationship or cascade error, the challenge of GRN prediction is increasing with the availability of data that have the total number of experimental observations very less compared to the number of genes. Reference [11] in his study stated that the total number of observations that are less than the number of genes in the experimental dataset has made the estimation unable to be performed by determining the weights to the whole set regulator (regulators). If the complete regulator set in a GRN is unable to be used in the calculation, some method has to be implemented to figure out the best way to use only some parts of the genes in calculation and at the same time does not affect the overall GRN prediction. Reference [9] conducted experiments on data with the number of nodes of 4,511 and 805 the number of observations. The lack of the total number of observations leads [9] to following a DREAM5 protocol that focuses only on correlation that happened in 141 transcription factors.

**Figure 1:**
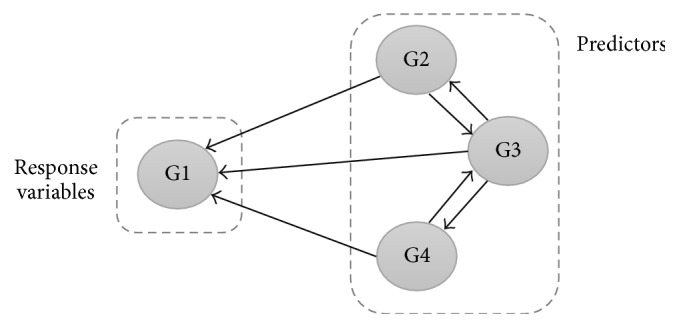
MLR in the context of GRN. MLR predicts the variations in the response variables from the variations in the predictors.

Regression analysis is a technique for modeling the relationships between two (or more) variables [40]. The Multiple Regression analysis models allow one to test several predictor variables that may explain different attributes about the response variables. Though complex, one can test all the factors that one thinks have an effect on a given response variable. This is unlike other inferior models that allow for only one predictor variable. Moreover, with the use of several variables, the accuracy of prediction is also improved. The terms* dependent variables*,* response variables*, and others have been used in the existing regression literatures interchangeably. The explanation on the meaning of each term, as well as the terms used throughout this manuscript is given in this section. Dependent variables are also known as* response variables* or* target variables*. As for independent variables, it is also known as* regressors* or* predictors *[41]. In order to ensure the consistency of the document, the terms* response variables* and* predictors* are used in the entire manuscript. GRN represents the scenario where the predictor variables are likely to be correlated with each other and they could all influence the response variables. Moreover, questions, such as how can we determine which variables are significant and how large of a role does each one variable play, do arise. All these questions can be answered by using the regression analysis. Thus, the scenario of MLR in the context of GRN is illustrated in Figure 1.

**Figure 2:**
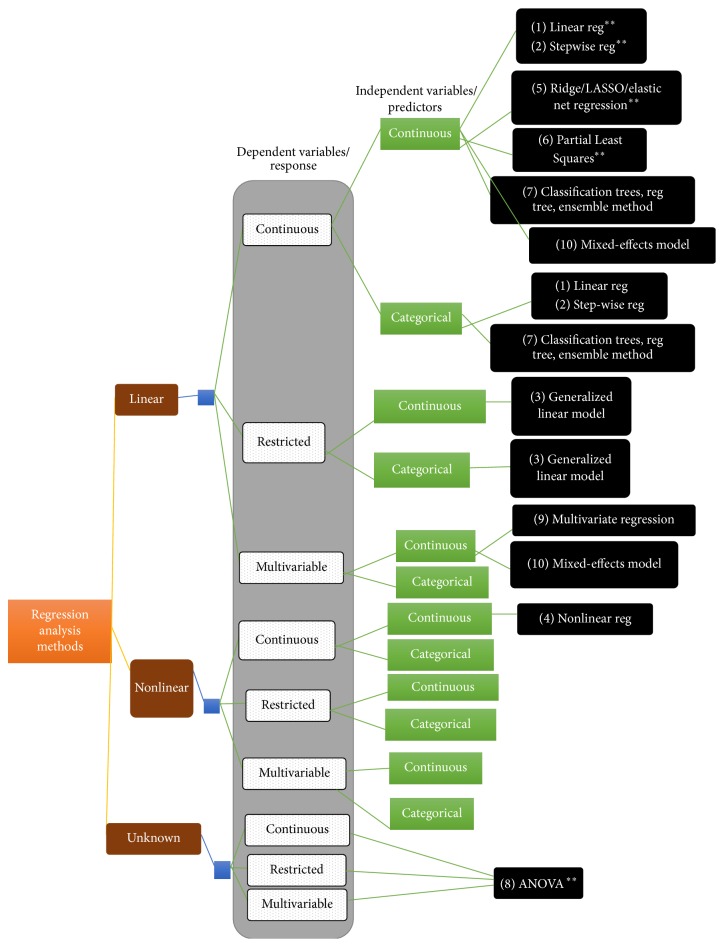
Regression analysis methods. The methods listed on the right are the recommended models extracted from Table 1. The methods that had been applied by other researchers are marked with double asterisks (*∗∗*).

We need to consider more than 10 regression-based methods before identifying the one that suits our experiments data. For that purpose, we categorize the regression analysis based on types of variables that each of the regression methods can handle. The categorization is based on our study in the literature study of the theory of regression analysis [39, 41, 42]. The categorization tree is shown in Figure 2. We produce the categorization tree to narrow down our method identification process. Nonlinear models represent the relationship between a continuous response variable and one or more continuous predictor variables.

The determination of the appropriate regression methods to be applied is highly dependent on how the researchers define the context of their GRN data. This is because each study involves data types and different research objectives. Apart from using the decision tree that we produced in Figure 2 as a basic guide, we use another decision tree shown in Table 1, presented by [43] for identifying the regression function. We redraw the decision tree in a more understandable form to facilitate the understanding of the possible model that can be used for data.

Referring to Table 1, we assume that interactions among the genes are described by the linear model [12, 44] due to the linear interaction between the response and independent variables. When identifying the type of response variable, either continuous or restricted or multivariable, we classify ours as continuous. Multivariable is a condition where multivariate regression may need to be applied. As for the type of independent variables, continuous type is more suitable.

## 6. Methods

Multiple regression takes into account the correlations between predictor variables and assesses the effect of each predictor variable, when other variables are removed [40]. On the other hand, linear regression uses one predictor variable to explain and/or to predict the outcome of response variables, while Multiple Regression (MLR) uses two or more predictor variables to predict the outcome [45] or, in other words, the response variable is influenced by more than one factor. In fact, MLR had been found to be the most suitable as it fits the nature of GRN with multiple genes that could cause multiple other genes to be activated [46]. In general, the response variable* y* may be related to *k* predictor variables. The general form of MLR with *k* regressor or predictor variables is shown in the following formula:

(1) yiβ0+β1Xi1+β2Xi2+⋯+βkXik+εi,=β0+∑j=1kβjxij+εi,i=1,…,n,

where *y*_*i*_ is *i*th observed response and *β*_*k*_ is *k*th coefficient and where *β*_0_ is the constant term/intercept in the model, *X*_*ij*_ is *i*th observation or level of regressor *x*_*j*_, and *ε*_*i*_ is *i*th noise term/random error.

The results of the program return a linear model of the responses* y*, fit to the data matrix *X* (observations on predictor variables). The predictor variables are specified as an* n*-by-*p* matrix, where *n*  is the number of observations, while *p* is the number of predictor variables. Each column of *X* represents one variable, and each row represents one observation. The response variable (*y*) is specified as an* n*-by-1 vector, where  *n*  is the number of observations. Each entry in *y* is the response for the corresponding row of *X*. The* least squares* technique had been applied to fit the model to the data. This method is the best when one is reasonably certain of the form of the model and mainly needs to determine the parameters [43]. The program was written using Matlab to apply the algorithm. Meanwhile, the programs that performed other major operations, such as extracting the results, assessing the performance and all processes pertaining to the experiment, were written in other separate files using Excel with embedded macro. All tests were performed on Intel Core with 3.20 GHz and 8 GB main memory that ran under the Windows 7 64-bit operating systems.

The predictors with rather high *p* values indicated that they might be unnecessary. The reported* p* value for predictors that were extremely small (less than 0.05) had been identified as the predictors that were used to create the response data. Why had *p* value less than 0.05 been chosen as the cut-off value? In statistics, the *p* value is a function of the observed sample results that is used for testing a statistical hypothesis. The *p* value is derived from the* t* statistics under the assumption of normal errors [47]. Before the test is performed, a threshold value is chosen, called the significance level of the test, traditionally 5% or 1% [48]. Statistical significance (or a statistically significant result) is attained when a *p* value is less than the significance level. Sharing the same opinion with [48, 49] states that as a matter of good scientific practice, a significance level is chosen before data collection and is usually set at 0.05 (5%). This fact is also supported by [50] who suggested that “a confidence interval is associated with a degree of confidence such as 0.95 (or 95%).” 95% means within 2 standard deviations of mean. Each observation in the datasets was taken into account when assessing the effect of each of the response variables.

## 7. Detecting the Cascade Motifs

The experiment that assesses the performance of MLR in predicting GRN was extended to assess the effectiveness of MLR in dealing with cascade error by using a novel experimental procedure proposed in this work. This section explains how the cascade motifs are detected. The list of the identified cascade motifs were used to assess the prediction performance.

The terms* cascade motifs* and* cascade errors* are used throughout the entire document.* Cascade motif* is defined as the edges that are identified by using certain methods to represent the condition where A → B → C, whereas* cascade error* is defined as an incorrect prediction of “shortcuts” or indirect interaction misinterpreted as direct interaction, where, in the case of A → B → C, the prediction always makes wrong prediction by predicting A → C [9, 35]. Moreover, the terms “directed edges,” “network,” and “node” are used in this manuscript to represent the terms “arcs,” “graph,” and “vertex,” which also present the same meanings but are more commonly used for discussion in the mathematics area. Note that the term “motif” has been used in other contexts to represent small connected subnetworks that occur in significantly higher frequencies than in random networks [51].

Additionally, the measures taken by [52] in GNW development (specifically network-motif analysis) were the most relevant reference in detecting the cascade motifs task in this study. The difference between the proposed method and the GNW in network motifs analysis had been that GNW engaged prediction confidence. GNW defined the prediction confidence of edges as their rank in the list of edge predictions. Besides, GNW scaled the prediction confidence such that the first edge in the list possessed confidence at 100%, while the last edge in the list had confidence at 0% [53].

Another notable difference between GNW and this research is that GNW analyzed all types of motifs, whereby the first step was definitely identification of all three nodes motif instances in the target network. Reference [52] used the algorithm proposed by [51] for this purpose. Nonetheless, since the focus of this study had been on cascade motifs alone, the researchers had been very much interested in working with the networks with directed edges and hence eliminated the need to identify three nodes motif instances in the large target network. Moreover, if determining prediction confidence of motif edges is treated as an important component in GNW, this study is different in such a way that identifying the cascade motifs had been concentrated as part of the target network. Furthermore, the method proposed would only be efficient for the small motifs, 3 nodes. This is because the applicability of the network motifs detection algorithm was never tested upon larger motifs.

**Figure 3:**
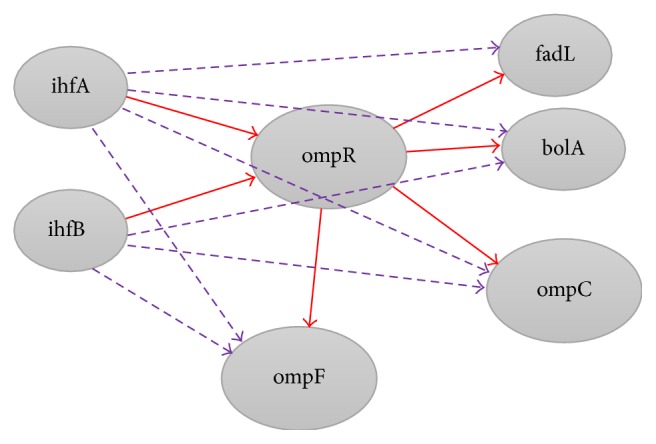
Cascade motifs in Table 2(b). The dashed lines show the cascade errors.

On top of that, in order to explain how cascade motifs were extracted from the GRN of* E. coli*, first, one needs to identify the directed size 3 subgraphs. More insights for the structure of DCE (Detecting Cascade Error) are provided via visualization shown in Table 2 and Figure 3. The following discussion uses some graph theoretic terminologies. Referring to Table 2, given a network *G* = (*V*, *E*), the edges of this graph all are directed and have been determined. As seen in Table 2(a), all the nodes in *V* are divided into two columns:* Col_One* and* Col_Two*. Referring to Table 2(b), the genes in* Col_One* are actually the regulators, while the genes in* Col_Two *are the target genes. Besides, there are* m* directed edges, where *m* = 1,2, 3,….

**Algorithm 1 (DCE).**

 

*Input*. A directed graph

*Output*. A list of cascade motifs

For each of the directed edges* m*, find the node that exists in both Col_One and Col_Two (node *V*_BOTH_)
Take note that for the directed edge, *V*_COL_ONE_→*V*_COL_TWO_ ≠ *V*_COL_TWO_→*V*_COL_ONE_
Eliminate the directed edges that do not contain *V*_BOTH_
Exclude *V*_BOTH_, pair each of the *V*_COL_ONE_ with each of the *V*_COL_TWO_

As depicted in Figure 3, the interactions with ompR as both regulator and target gene had been extracted.

**Figure 4:**
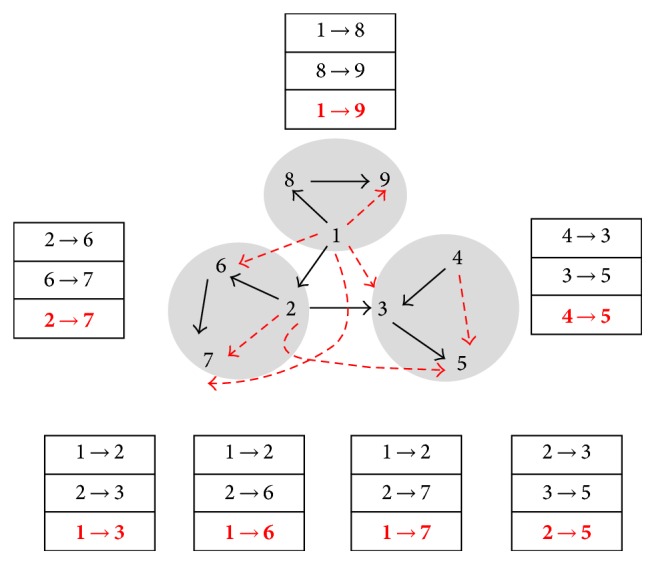
List of directed edges and cascade motifs. The numbers represent the name of genes. The dashed arrows represent cascade errors, while the black texts represent the cascade motifs.

Figure 4 presents the application of DCE when many subgraphs were involved.

## 8. Extracting Subnetworks

Since the number of observations of the real experiment datasets was far less than the number of predictors, some predictors were eliminated systematically by extracting the random subnetworks from global interaction networks using the established subnetwork extraction method proposed by [53]. The following paragraph explains how the subnetworks are extracted.

There are numerous rules of thumb for the number of observations needed per predictor variable. Reference [54] suggested 10 observations for each predictor variable. In the case of this study, since the experiment involved 4297 number of genes, the number of observations should be 42,970. Besides, as the maximum number of observations in M3D was only 907, it was impossible for the MLR to be employed for all the 4297 genes. Reference [41] suggested to eliminate some predictors in order to solve problems related to limited observations. With that, we propose the predictors in the datasets to be eliminated by extracting the subnetworks from the global datasets, where each of the subnetworks consisted of less than 907 number of genes. For all the three subnetworks used in this experiment, the parameter seed was set to random vertex, while the neighbor selection was set to random among top 10%. Random vertex seed means, for each subnet, the extraction method starts from a different randomly picked seed node of the source network. Setting some percentage for neighbor selection will allow for tuning of the sampling strategy from pure modular subnetwork extraction to random subnetwork extraction [34]. This setting adds some stochasticity to the subnetworks as well.

## 9. Results of the Experiments 

An experiment to assess the general performance of MLR was conducted prior to the experiment that studied the performance of MLR in predicting cascade motifs. The precascade motifs experiment was conducted to ensure that the proposed model could at least achieve the acceptable range of AUROC. In this work, where real complex data were involved, AUROC ≥ 0.5 had been regarded as to achieve the acceptable standard [55].  Table 3 shows the results of using the datasets in natural settings, which means that the cascade motifs were excluded on purpose.

Different subnetworks were tested to prove that, even with different group data, the results had been consistent. These subnetworks consisted of different network sizes, where the extraction process has been described in Section 8. The results show that all testing obtained AUROC > 0.5, which conceded the researchers to further investigate the effects to the cascade motifs.  Table 4(a) shows that, out of 1348 cascade motifs in set 1, only 10 errors (0.74%) are due to the cascade error. Set 2 does not contain any cascade error.  Table 4(b) shows that Set 3 contains 94 cascade errors (7%) out of total 1348 cascade motifs. From the results, it can be concluded that wrong predictions due to cascade errors were very minimal, where only two subnetworks have cascade errors and the amount is less than 10% for both subnetworks.

Table 5 displays the AUROC values of the same experiments that the results are shown in Tables 4(a) and 4(b). The AUROC values for all the three experiments had been greater than 0.5, hence achieving an acceptable minimum and surpassing the achievements of a GRN prediction method [55]. Compare the two scenarios where (1) the prediction is made on the entire* E. coli* experimental data and (2) the prediction is applied to the subnetworks containing cascade motifs. If the number of GRN relationships in the target networks is 1000 and the number of wrong predictions is 10, the percentage of correct predictions is 99%. Compared to the second scenario (the experiment applied in this work), with the small number of GRN relationships, for example 100, even though the number of wrong predictions is similar to the scenario (1), the percentage of correct prediction is 90% only. Due to the large difference in terms of the number of genes predicted, the results of our experiments could be said to have achieved the acceptable level of performance. With the size of the subnetworks involved in our experiments being 5 times less than the size of a large network of* E. coli*, it is reasonable that the acceptable level is assumed to be AUROC > 0.5.

Each of these datasets contains different percentage of cascade motifs (refer to Tables 4(a) and 4(b)) and different number of possible edges (refer to Table 5). The mixture of complexity level of all these datasets results in a small difference between AUROC values achieved by all these three datasets. The narrow range and the consistent AUROC values recorded by all three experiments proved that the results of this experiment reflected the overall ability of prediction methods proposed in this project in resolving cascade errors. Three sets used in this experiment were carried out to ensure that the study covered various subnetworks of a large network of* E. coli*.

## 10. Comparison with the Other Methods

Table 6 shows the comparison of the method proposed with other 6 selected methods, where the results were reported by [10].

Compare the two scenarios where (1) the prediction is made on the entire* E. coli* experimental data and (2) the prediction is applied to the subnetworks containing cascade motifs. If the number of GRN relationships in the target networks is 1000 and the number of wrong predictions is 10, the percentage of correct predictions is 99%. Compared to the second scenario (the experiment applied in this work), with the small number of GRN relationships, for example 100, even though the number of wrong predictions is similar to the scenario (1), the percentage of correct prediction is 90% only. Thus, the experimental results of this project could be said to be highly comparable with other methods. This is because other methods of conducting experiments like scenario (1) indeed tend to produce positive results, compared to the prediction generated in this study. Moreover, with the size of the subnetworks involved in the experiment being 5 times less than the size of a large network of* E. coli*, it is reasonable that the obtained AUROC value was slightly lower than that of other methods.

## 11. Collinearity Diagnostics Test

Multicollinearity is a serious problem that may dramatically affect the usefulness of a regression model [41]. The existence of high correlations among the independent variables in a regression model is known as multicollinearity [62]. Moreover, there are various methods for diagnosing multicollinearity, such as observing the values of Variance Inflation Factors, Variance Proportions, and Principal Components [62]. Eigensystem analysis [41] and Belsley collinearity diagnostics test are added to the list of diagnostics [61]. In this study, the Belsley collinearity test was employed to determine the degree of multicollinearity in the datasets. The program was run by using Matlab. Furthermore, [61] recommended that the sources of collinearity to be diagnosed are (a) only for those components with large CI and (b) for those components for which VDP (variance decomposition proportions) is large (say, VDP > 0.5) on two or more variables. Besides, numerical experiments by [61] indicated that the following ranges (Table 7) are useful.

Table 8 shows sample of diagnostic test data. With the Belsley method, more than 90% of the components exhibited CIs greater than 100, indicating that the collinearity affected the data severely. However, none of the VDPs had been associated with all the large CIs that displayed values less than 0.5. Moreover, more information was sought from [62] where they asserted that the multicollinearities in the data appear to involve almost all variables when there is no large variance proportion or VDP for the large CIs.

With the Belsley method, more than 90% of the components exhibited CIs greater than 100, indicating that the collinearity affected the data severely. However, none of the VDPs had been associated with all the large CIs that displayed values less than 0.5. Moreover, more information was sought from [62] where they asserted that the multicollinearities in the data appear to involve almost all variables when there is no large variance proportion or VDP for the large CIs.

## 12. Analysis

Successful use of the mathematical model to solve problems in biological sciences requires understanding of the theoretical underpinnings of the phenomena, the statistical characteristics of the model, and the practical problems that may be encountered when using these models in real-life situations. Multiple Linear Regression (MLR) is a well-known statistical method based on ordinary least squares regression. This operation involves a matrix inversion, which leads to collinearity problems if the variables are not linearly independent.

After applying MLR in this work, we identify several limitations of MLR such as being unable to handle issue of collinearity between independent variables (predictors), being unable to cater for *n* ≤ *p* datasets, and MLR dealing with only one response variable at a time. The good model for GRN inference should handles several responses simultaneously. In MLR, the observed response values are approximated by a linear combination of the values of the predictors. The coefficients of that combination are called regression coefficients or *B*-coefficients. In case of collinearity among predictors, the *b*-coefficients are not reliable and the model may be unstable. MLR also tends to overfit when noisy data is used.

The practical problems most often encountered in regression analyses are outliers and influential observations, and multicollinearity, as well as a model with extraneous variables [41, 62]. The results of Belsley collinearity test performed in this experiment proved that multicollinearity had affected the datasets greatly. In the context of GRN, the most relevant source of multicollinearity is the issue of an overdefined model. An overdefined model has more regressor variables than observations. The overdefined model is always encountered in biology experiments, where there may be only a small number of subjects available, and information is collected for a large number of regressors on each subject. Reference [41] pointed out three specific recommendations to eliminate some of the regressors: (1) redefine the model in terms of a smaller set of regressors, (2) perform preliminary studies by using only subsets of the original regressors, and (3) use principal-components-type regression methods to decide which regressors to remove from the model. As demonstrated by our works, we eliminated some of the regressors by extracting the subnetworks using a systematic approach (with the help of tool named GNW). However, this approach requires additional study that has to be conducted to ensure that the interrelationships between the regressors are not ignored.

## 13. Conclusion and Future Direction

This research proposed an algorithm for reconstructing GRN with the main aim to solve cascade error problem. Nonetheless, this work differed from other manuscripts that have been widely published in a way that it presented novel experimental procedures to assess the effectiveness of GRN inference method in dealing with cascade error. Besides, from a detailed research on the nature and the source of the data under study, regression analysis was chosen because it establishes objective measures of relationships between the predictor and the response variables. Based on the study of all the regression techniques, MLR has been identified as the most suitable method to solve the cascade errors because it takes into account the combination of effects and simultaneous observations. The resulting* p* value for predictors that had been less than 0.05 was identified as the predictors that were used to create the response variables. This study also evaluated the performance of MLR in predicting the 3-node motifs. For path 1 → 2 → 3 as an example, the occurrences of false prediction that suggested the existence of a direct link between them (1 → 3) had been investigated. The experiment further revealed that the number of cascade errors was very minimal at 2 out of 3 tested subnetworks. Despite the multicollinearity problem and limited observations data, satisfactory results had been achieved as all the tested subnetworks attained AUROC values above 0.5.

MLR involves a matrix inversion, which leads to collinearity problems if the variables are not linearly independent. The nature of GRN predictors is in contrast with the requirements of MLR. For MLR, the ability to vary independently of each other is a crucial requirement to variables used as predictors. MLR also requires more samples than predictors or the matrix cannot be inverted.

With regard to our experiment, even though MLR appears to be able to handle cascade errors, the identified limitations detected in MLR make us recommend that other regression technique shall be used to replace MLR with GRN inference, particularly when *n* ≤ *p* type of datasets is involved. Even though we have tried to eliminate some of the predictors using a systematic approach (as proposed in this work), that method requires more detailed study on how to combine prediction on separated subnetworks to represent the whole* E. coli* networks.

## Figures and Tables

**Table 1:** Decision tree.

Nature of data								Chosen model type	Recommended model
	Independent variables (predictors)		Dependent variables (response variables)			Model			
	Continuous	Categorical	Continuous	Restricted	Multivariable	Linear	Nonlinear		
(1)	*✓* (OR)^*∗*^	*✓* (OR)	*✓* ^*∗*^			*✓* ^*∗*^		Fitted model coefficients	^*∗∗*^Linear regression
(2)	*✓* (OR)^*∗*^	*✓* (OR)	*✓* ^*∗*^			*✓* ^*∗*^		Fitted model and fitted coefficients	^*∗∗*^Stepwise regression
(3)	*✓* (OR)	*✓* (OR)		*✓*		*✓* (generalized)		Fitted generalized linear model coefficients	Generalized linear models
(4)	*✓* ^*∗*^		*✓* (nonlinear)^*∗*^				*✓* ^*∗*^	Fitted nonlinear model coefficients	^*∗∗*^Nonlinear regression
(5)	*✓* ^*∗*^		*✓* ^*∗*^			*✓* ^*∗*^		Ridge/LASSO/elastic net regression	^*∗∗*^Ridge/LASSO/elastic net regression
(6)	*✓* (correlated)^*∗*^		*✓* ^*∗*^			*✓* ^*∗*^		Fitted model and fitted coefficients	^*∗∗*^Partial Least Squares
(7)	*✓* (OR)^*∗*^	*✓* (OR)	*✓* ^*∗*^					Nonparametric model	^*∗∗*^Classification trees, Regression trees, Ensemble methods
(8)		*✓*						ANOVA	ANOVA
(9)	*✓*				*✓*	*✓*		Fitted multivariate regression model coefficients	
(10)	*✓*		*✓*		*✓*	*✓*		Fitted mixed-effects model coefficients	Mixed-effects model

Note: cells with “*∗*” indicate the type of variables that suit the nature of GRN. The recommended models are marked with asterisks (*∗∗*).

**(a):** 

*Col_One*	*Col_Two*
hupB	tyrP
crp	hupA
narL	dmsB
narL	dmsC
ihfA	ompR
ihfB	ompR
ompR	fadL
ompR	bolA
ompR	ompC
ompR	ompF

**(b):** 

*Col_One* *Regulator*	*Col_Two* *Target Gene*
ihfA	ompR
ihfB	ompR
ompR	fadL
ompR	bolA
ompR	ompC
ompR	ompF

**Table 3:** Results of the experiments that used the datasets in which the cascade motifs have been removed.

	Number of genes	Number of observations	*p* value	AUROC
Subnetwork A size 415	415	466	<0.05	0.6860
Subnetwork A size 415	415	466	<0.04	0.6795
Subnetwork A size 415	415	907	<0.05	0.6622
Subnetwork B size 893	893	907	<0.05	0.5022
Subnetwork C size 871	871	907	<0.05	0.5081

**(a):** 

Case	Total cascade motifs	Total number of “cascade motifs” that match with GS TRUE_CASCADE	Multiple Linear Regression *total number of incorrect prediction due to “cascade errors”* *CASCADE_ERR*	Percentage of cascade motifs in datasets
Set 1				
gadE	105	29	3	0.16%
csgD	41	12	0	
arcA	157	77	0	
gadX	216	53	3	
dcuR	21	15	0	
marA	150	40	1	
fis	658	173	3	

*Total*	*1348*	*399*	*10*

Set 2				
gadE	105	29	0	0.12%
csgD	41	12	0	
arcA	157	77	0	
gadX	216	53	0	
dcuR	21	15	0	
marA	150	40	0	
fis	658	173	0	

*Total*	*1348*	*399*	*0*

**(b):** 

Case	Total cascade motifs	Total number of “cascade motifs” that match with GS TRUE_CASCADE	Multiple Linear Regression *total number of incorrect prediction due to “cascade errors” CASCADE_ERR*	Percentage of cascade motifs in datasets
Set 3				
gadE	105	29	3	0.54%
csgD	41	12	0	
arcA	157	77	14	
gadX	216	53	8	
dcuR	21	15	5	
marA	150	40	7	
fis	658	173	57	

*Total*	*1348*	*399*	*94*

^*∗∗*^Note:

(1) Percentage of cascade motifs in datasets ((Total cascade motifs – Total TRUE_CASCADE)/Total number of possible edges) × 100.

(2) Refer to Table 4 for the total number of possible edges.

(3) Cascade motif is defined as A → C for the case of A → B → C.

**Table 5:** Characteristics of the datasets tested in the experiment and the AUROC results.

	Set 1	Set 2	Set 3
Number of *cascade motif genes* in the tested datasets	363	360	160
Number of *random genes* in the tested datasets (not redundant with cascade motif genes)	397	533	255
Total number of tested genes	760	893	415
Total number of possible edges	576,840	796,556	171,810
Total number of correct prediction (CORRECT_PRED)	119	10	825
Total number of incorrect prediction	27,526	253	170,985

*AUROC*	0.511	0.502	0.662
	*Average: 0.5584 *		

^*∗∗*^Note:

(1) Cascade motif genes (italic text) are referring to the gene itself. This is not similar to cascade motif.

(2) Number of cascade motif genes in the tested datasets are obtained by comparing the cascade motifs genes with the genes in the datasets.

(3) Total number of possible edges = Total number of tested genes × (Total number of tested genes – 1).

**Table 6:** AUROC of selected methods on the M3D datasets of *E. coli*.

Methods	References	M3D	Experimental data
ANOVA^*∗*^	[10]	0.798	One whole network of* E. coli*
Genie3^*∗*^	[56]	0.673	
Pearson^*∗*^	[57]	0.646	
MRNet^*∗*^	[58]	0.645	
CLR^*∗*^	[59]	0.642	
ARACNe^*∗*^	[60]	0.635	

MLR	This article	0.558	Predetermined subnetworks that consist of expression data with added cascade motifs

Note: the results marked in *∗* are reported by [10].

**Table 7:** CI and the level of collinearity [61].

Condition index (CI)	Collinearity
5 < CI < 10	Weak
30 < CI < 100	Moderate to strong
CI > 100	Severe

**Table 8:** The CIs and the VDPs of four genes from Set 3 as example of data generated from the diagnostic test.

condIdx	aaeA_b3241_14	aceA_b4015_15	aceE_b0114_15	aceF_b0115_15
		…		
168.1754	0	0.0003	0	0
172.9103	0	0.0001	0.0001	0.0001
176.3094	0	0.0001	0.0001	0.0001
176.8486	0	0.0002	0	0
182.4254	0	0.0002	0	0
		…		
